# Ocular Accommodative and Pupillary Responses During Fixation on Augmented Reality With a Maxwellian Display

**DOI:** 10.1167/iovs.65.11.30

**Published:** 2024-09-18

**Authors:** Masakazu Hirota, Kakeru Sasaki, Kanako Kato, Ryota Nakagomi, Ryusei Takigawa, Chinatsu Kageyama, Seiji Morino, Makoto Suzuki, Toshifumi Mihashi, Atsushi Mizota, Takao Hayashi

**Affiliations:** 1 Department of Orthoptics, Faculty of Medical Technology, Teikyo University, Itabashi-ku, Tokyo, Japan; 2 Department of Ophthalmology, School of Medicine, Teikyo University, Itabashi-ku, Tokyo, Japan; 3 Graduate Degree Program of Health Data Science, Teikyo University, Itabashi-ku, Tokyo, Japan; 4 Graduate Degree Program of Comprehensive Applied Data Science, Teikyo University, Itabashi-ku, Tokyo, Japan; 5 QD Laser, Kawasaki-ku, Kanagawa, Japan; 6 Nishikasai Inouye Eye Hospital, Edogawa-ku, Tokyo, Japan

**Keywords:** augmented reality, Maxwellian display, accommodation, pupils, ocular refraction

## Abstract

**Purpose:**

This study aimed to investigate the changes in ocular refraction and pupillary diameter during fixation on augmented reality (AR) images using a Maxwellian display.

**Methods:**

Twenty-two healthy young volunteers (average age, 20.7 ± 0.5 years) wore a Maxwellian display device in front of their right eye and fixated on an asterisk displayed on both a liquid-crystal display (real target) and a Maxwellian display (AR target) for 29 seconds (real as a baseline for 3 seconds, AR for 13 seconds, and real for 13 seconds) at distances of 5.0, 0.5, 0.33, and 0.2 meters. A binocular open-view autorefractometer was used to measure the ocular refraction and pupillary diameter of the left eye.

**Results:**

Accommodative (5.0 meters, 0.28 ± 0.29 diopter [D]; 0.5 meter, −0.12 ± 0.35 D; 0.33 meter, −0.43 ± 0.57 D; 0.2 meter, −1.20 ± 0.82 D) and pupillary (5.0 meters, 0.07 ± 0.22 mm; 0.5 meter, −0.08 ± 0.17 mm; 0.33 meter, −0.16 ± 0.20 mm; 0.2 meter, −0.25 ± 0.24 mm) responses were negative when the real target distances were farther away. The accommodative response was significantly and positively correlated with the pupillary response during fixation on the AR target (*R*^2^ = 0.187, *P* < 0.001).

**Conclusions:**

Fixating on AR images using a Maxwellian display induces accommodative and pupillary responses. Accommodative responses depend on the distance between real objects. Overall, the Maxwellian display does not completely eliminate accommodation in real space.

Augmented reality (AR) is an innovative technology that supplements the real-world environment with computer-generated sensory inputs, such as visual, auditory, or haptic information. These virtual components appear to coexist with real objects in the same space, enhancing the user's perception of reality and enriching the information content provided.^1^ According to Azuma,^2^ AR can be described by three key features: a combination of real and virtual objects in a real environment; real-time interaction with the system, which is capable of reacting to the user's inputs; and geometrical alignment of virtual objects to physical objects in real space. AR technology has numerous applications in various fields, including education, entertainment, medicine, and industrial settings.^3^ For example, AR systems can provide valuable support to technicians by displaying relevant information, such as equipment parts, maintenance steps, and instructions, directly in their field of view.^4^^–^^7^ This can lead to improved efficiency, accuracy, and safety in maintenance operations. The use of AR has also recently advanced in the field of medicine^8^; for example, visualization of the area of a lesion via real-time AR assists the surgeon and improves surgical efficiency.

However, the long-term or continuous use of AR devices can cause discomfort to the user.^9^ In particular, vergence accommodation conflict (VAC) is a problem that can lead to visual fatigue and dizziness.^10^^–^^14^ VAC is a physiological phenomenon observed when viewing stereoscopic or virtual images in which convergence is focused on the virtual image and accommodation is focused on the display (real objects).^10^^,^^15^^,^^16^ To avoid VAC, some displays have been equipped with a Maxwellian view (i.e., Maxwellian displays).^12^^,^^17^^,^^18^ The Maxwellian view presents an image to the retina by converging rays at the eye pupil and provides in-focus images, regardless of the refractive error of the human eye.^19^^,^^20^ Users can see a relatively clear image, with a mean visual acuity of 0.50 logarithm of the minimum angle of resolution (logMAR) during unassisted vision, independent of refractive errors.^21^

Maxwellian displays are less affected by refractive error because the light beam is focused on the pupil, thus reducing the effective pupillary diameter and increasing the depth of focus.^12^^,^^17^^,^^18^ The extended depth of focus provides an expanded range of clear vision, and Maxwellian displays are theoretically considered accommodation free.^22^ However, AR images are superimposed on real objects, which is expected to induce accommodative responses with reference to the position of the real object.^23^ Thus, we hypothesize that the accommodative response cannot be completely eliminated when Maxwellian displays are used in real space.

Given that both accommodative and pupillary responses are controlled by the parasympathetic nervous system,^24^ concurrent measurements of ocular refraction and pupillary diameter can provide insights into the potential impact of AR displays on human vision. Therefore, this study aimed to simultaneously evaluate changes in ocular refraction and pupillary diameter during fixation on AR images through wearable glasses equipped with a Maxwellian display.

## Methods

### Participants

In this study, a difference (delta) was defined as a change in the ocular spherical equivalent refraction (SER) of ≥0.25 diopters (D) before and after use of the Maxwellian display. The SER is calculated using the following equation:

$$\begin{array}{c} \mathrm{SER}\;=\;S+\frac{C}{2} \end{array}$$

where *S* and *C* are the spherical and cylindrical lens powers, respectively. A cylindrical lens was used only in the negative format. The data variance (sigma) was set to 0.25 D considering the accommodation fluctuation during the measurement. We set α at 0.05 and β strictly at 0.95. A sample size of 19 individuals was needed; considering a dropout rate of 15%, the sample size was set to 22 participants.

Twenty-two healthy young adult volunteers (mean age ± SD, 20.7 ± 0.5; range, 20–21 years; 20 females, two males) participated in this study. Participants were excluded if they had any ocular disease, a distance visual acuity worse than 0.0 logMAR, blurred vision at 0.2 meter, astigmatism greater than 2.0 D, or log stereoacuity worse than 2.0 log arcsec. All participants underwent complete ophthalmologic examinations, including the determination of best-corrected visual acuity at a distance of 5.0 meters, accommodation (blur point) in each eye using the push-up method (Ishihara's near point meter; Handaya Co., Ltd., Tokyo, Japan), near point of convergence, stereoscopic acuity at 40 cm (Titmus stereotest; Stereo Optical Company, Chicago, IL, USA), and heterophoria by using an alternating cover test at near (0.33 meter) and at distance (5.0 meters), as well as fundus examinations. Stereoacuity was converted into the logarithm of the arc second.

All participants provided informed consent after we explained the nature of this study and possible complications. The experimental protocol and consent procedures were approved by the Institutional Review Board of Teikyo University (approval no. 19-154-5). This investigation adhered to the tenets of the Declaration of Helsinki.

### Measurement of the Accommodative Response

#### AR Image Projection

**Figure 1.**
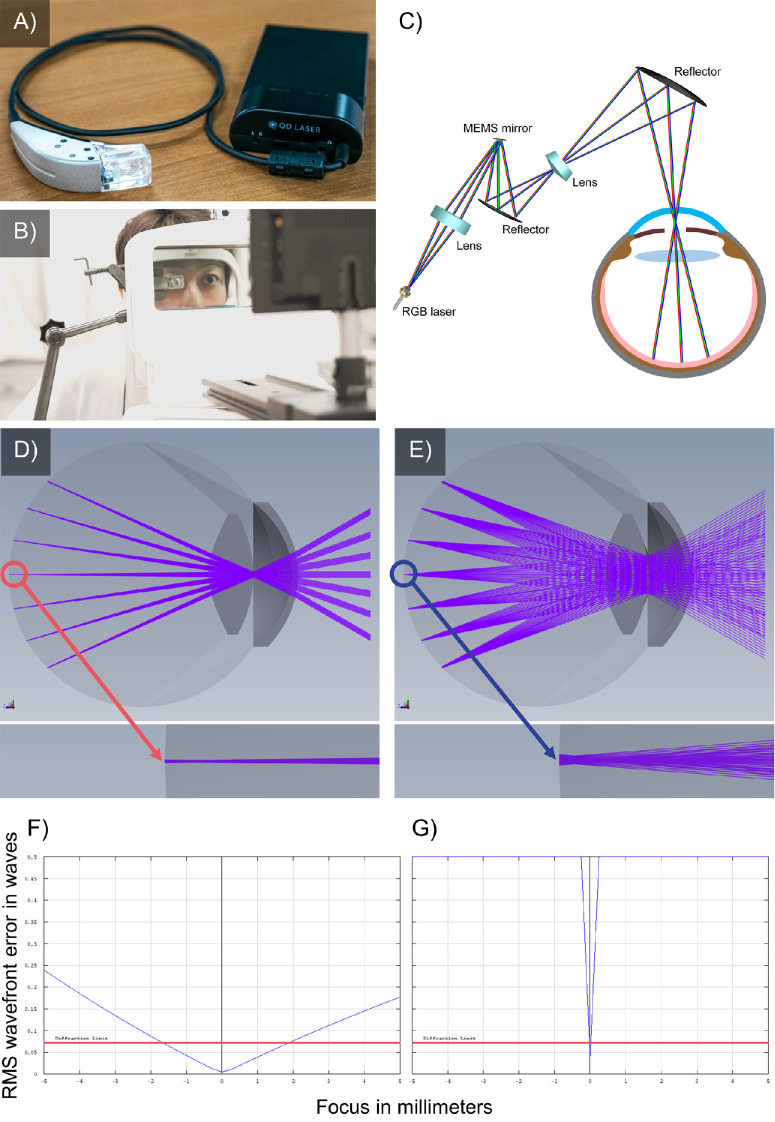
Overview of the RETISSA Display II. (**A**) The RETISSA Display II is a wearable Maxwellian display that projects an AR image. (**B**) In this study, the RETISSA was held on a flexible arm and set in front of the participante. on and discrimination pentnt set in frocould see the target on the RETISSA and the LCD. (**C**) The RETISSA uses three colors of a semiconductor laser (RGB laser) with a picoprojector, whereas the RGB laser is controlled via a MEMS mirror. (**D**, **E**) Optical diagram of the RETISSA (**D**) and sunlight (**E**) from the emmetropic human eye. The RETISSA has a narrower luminous flux than sunlight. Furthermore, the RETISSA has collimated light at all retinal positions. (**F**, **G**) Simulated depth of focus in the RETISSA (**F**) and in sunlight (**G**) in the emmetropic human eye. The *blue* *lines* and *red lines* indicate the simulated optical image quality and the diffraction limit, respectively. Negative and positive signs on the *x*-axis indicate myopia (negative) and hyperopia (positive), respectively. The depth of focus is greater in the RETISSA than in sunlight in emmetropic human eyes.

**Figure 2.**
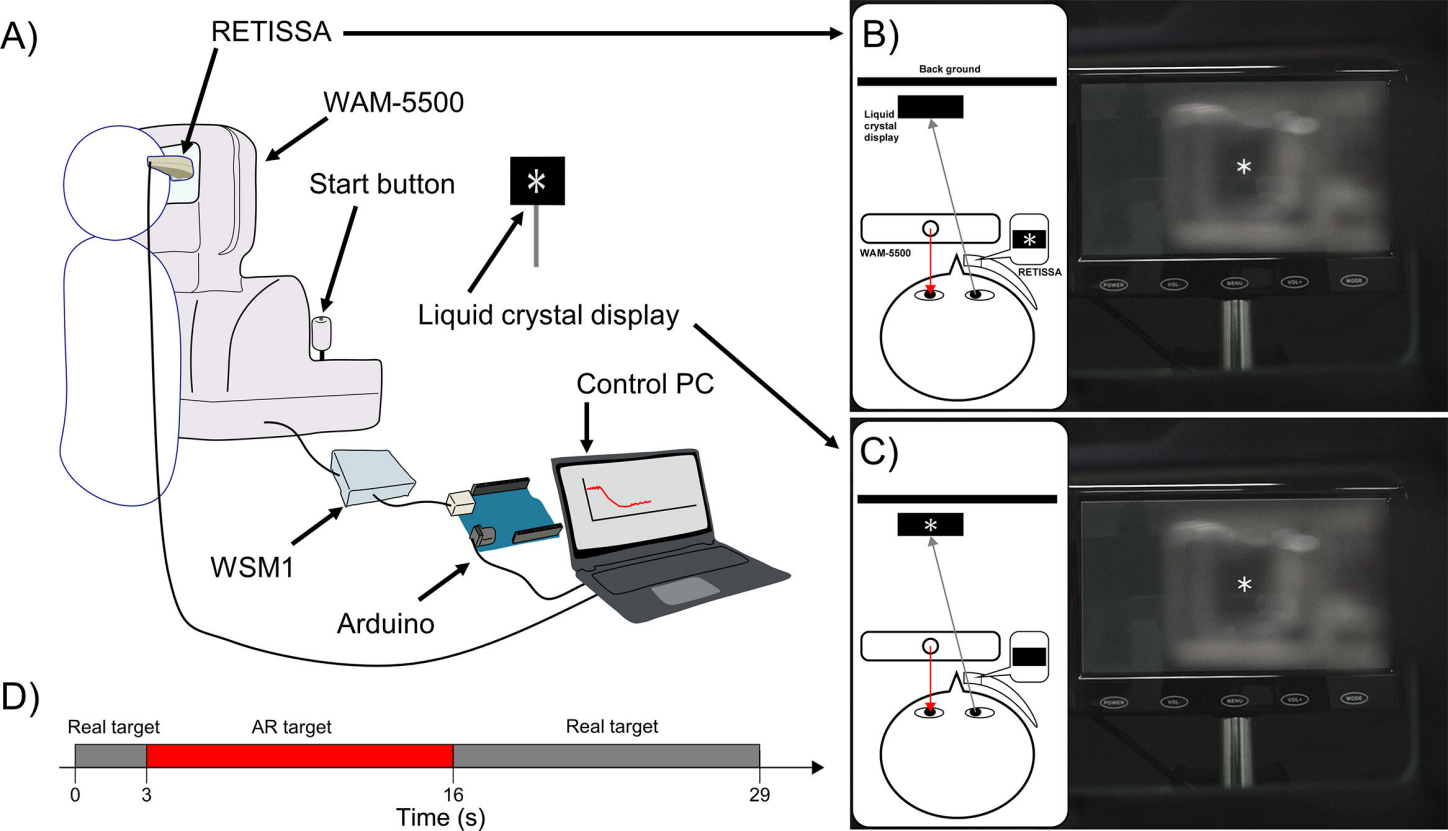
Experimental procedure. (**A**) The participants wore a Maxwellian display device (RETISSA Display II) on their right eye and fixated on an asterisk target displayed on both an LCD (real target) and a Maxwellian display (AR target) for 29 seconds. The AR image was presented to the right eye, and changes in the SER and pupillary diameter of the left eye were recorded by the WAM-5500. The real target was turned off when the AR image was presented, and the AR target was turned off when the real target was presented. (**B**, **C**) The *red* and *gray arrows* indicate the measurement light of the autorefractometer and the participant's gaze, respectively. (**D**) The *horizontal* *bars* and *red bars* indicate the duration of real-target presentation and the duration of AR target presentation, respectively.

We used the RETISSA Display II (QD Laser, Kanagawa, Japan) as a wearable Maxwellian display to project AR images (Fig. 1A). The RETISSA used three colors (red, green, blue [RGB]) of a semiconductor laser via a picoprojector, whereas the RGB laser was controlled by using a microelectromechanical systems (MEMS) mirror. The RGB laser light entered the eyes via a reflector. The RGB (red, 640 nm; green, 515 nm; blue, 465 nm) lasers can project parallel beams of light to the retina without any vignetting in the cornea or lens when the diameter of the RGB laser beam entering the cornea is 0.6 mm (Fig. 2B). The RGB laser power of the RETISSA (5.72 µW/cm^2^) satisfies the Class I laser criterion set by the International Electrotechnical Commission and is lower than the International Organization for Standardization criterion of 220 µW/cm^2^, which specifies the absence of retinal damage after 2 hours of irradiation with visible light.

The RETISSA was designed to be focus free by increasing the depth of focus to prevent defocusing, which occurs when the light rays do not converge correctly on the retina due to axial length or refractive errors. The RETISSA has a field of view of 30° under a pupillary diameter of 4.0 mm, and the resolution is 1280 × 720 pixels.

#### Binocular Open-View Autorefractometer

We used a WAM-5500 Autorefractometer (Shigiya Machinery Works, Hiroshima, Japan) in this study. The WAM-5500 is a binocular open-view autorefractometer that can simultaneously measure the SER and pupillary diameter of one eye with close to natural vision. Furthermore, the WAM-5500 enables continuous measurement of the SER and pupillary diameter with a sampling rate of 5.0 Hz when connected to a WAM-5500 status monitor (WSM-1; Shigiya Machinery Works). The SER measured by the WAM-5500 is output to the second decimal place, and the pupillary diameter is output to the first decimal place.

#### Fixation Targets

For the real target, the participant fixated on a white asterisk on the black background of a 7-inch liquid-crystal display (LCD; Itprotech International, Chiba, Japan). The distances of the real target were set at 5.0, 0.5, 0.33, and 0.2 meters in front of the left eye, and the visual angles were equivalent to a visual acuity of 1.91 logMAR for each distance (Supplementary Video S1). The luminance of the real and AR targets was set at 180 cd/m^2^, whereas that of the background was set at 2 cd/m^2^ using a BM-9A luminance meter (Topcon, Tokyo, Japan).

The AR targets were adjusted to the same size and luminance as those of the real target. The AR and real targets in the participant's line of sight were placed in parallel to prevent eye movements induced by target switching. The revealing and hiding of the AR target were controlled through a personal computer (control PC) running on a Windows operating system (Microsoft, Redmond, WA, USA) linked to the WAM-5500 and RETISSA through customized software written in Python 3.8.5.

#### Optical Adjustments

The Maxwellian display theoretically provides an accommodation-free state to its users.^19^ In the original Maxwellian view, light rays enter the center of the pupil, but the light rays could not be observed in this study due to equipment limitations. As an alternative, the target was displayed at the center of the Maxwellian display screen and adjusted to the subjective central visual field for all participants (Supplementary Videos S1 and S2). To avoid off-axis aberration caused by differences in line of sight (LOS) when participants fixated on two targets, the control LCD and asterisk projected by the Maxwellian display were both placed on the same straight line (Supplementary Video S2). Considering that the LOS approximates the visual axis,^25^ the Maxwellian view was considered established in this study. If the Maxwellian view is established, the depth of focus is expected to become deeper, and the visual acuity of the naked eye is expected to be approximately 0.3 logMAR, which is the resolution limit of the RETISSA, even in the case of high myopia. To further confirm the establishment of the Maxwellian view, the uncorrected visual acuity (UCVA) of the right eye was measured with the trial frame in real space and with the RETISSA being worn after completion of the optical setting in each participant.

#### Perceived AR Target Distance

If the optical theory of the Maxwellian view is correct, AR targets are always perceived as more distant than real targets. Moreover, if the accommodation is working, it is expected that the targets are at the same position or that the AR image is superimposed on the real object so that the position seems the same or closer than the real object. Thus, the participants were asked which of the AR or real targets appeared to be closer or in the same position after completing the examination at each distance.

### Experimental Procedures

The SER in all participants was measured using the WAM-5500, and the SER in both eyes was corrected using soft contact lenses (SCLs; SEED 1dayPure Moisture; Seed Co., Ltd., Tokyo, Japan). The examiner then confirmed the participant's SER at 5.0 meters. The SCL power was adjusted until the participant's SER over SCL wear was between −0.50 D and 0.00 D. The RETISSA was held by a flexible arm, and its position was adjusted to project the AR target to the participant's right eye (Figs. 1B, 2A; Supplementary Video S2) while simultaneously measuring the accommodative and pupillary responses in the nonviewing left eye.

When one eye is covered and the accommodative stimulus is presented to the viewing eye, accommodative and pupillary responses also occur in almost equal amounts in the nonviewing eye.^26^^–^^28^ This phenomenon is termed the consensual response. In this study, the SER and pupillary diameter of the left eye, which was not fixating on the AR target, were measured via a consensual response.

The SER and pupillary diameter of the left eye were continuously measured by the WAM-5500, and the real target was displayed for 3 seconds to establish a baseline. Then, the target was subsequently changed from the real target to the AR target for 13 seconds (Fig. 2B) and from the AR target to the real target for 13 seconds (Fig. 2C). As the pupillary diameter was dependent on illumination, the illumination of the room was set to 500 lux using an illuminometer (IM-2D; Topcon). All of the participants were asked to look at the center of the asterisk for 29 seconds during the examination (Fig. 2D). Each participant performed this step of stimulation five times for each distance. The targets were presented sequentially at 5.0, 0.5, 0.33, and 0.2 meters.

### Data Analysis

Data on the SER and pupillary diameter of the left eye were exported to a comma-separated values file. Data in which the pupillary diameter changed by more than 2 mm in a single frame were defined as blinks and treated as missing values.^29^ Then, the missing value was replaced with a linearly interpolated value calculated from an algorithm written with Python 3.8.5. For every participant, the SERs and pupillary diameters were measured five times each at four distances (5.0, 0.5, 0.33, and 0.2 meters). The five measurements of the SERs and pupillary diameters were averaged frame by frame for 29 seconds (146 frames in total) at each distance. The baseline SERs and pupillary diameters, while being fixed on the AR target and on the real target, were subsequently calculated individually.

**Figure 3.**
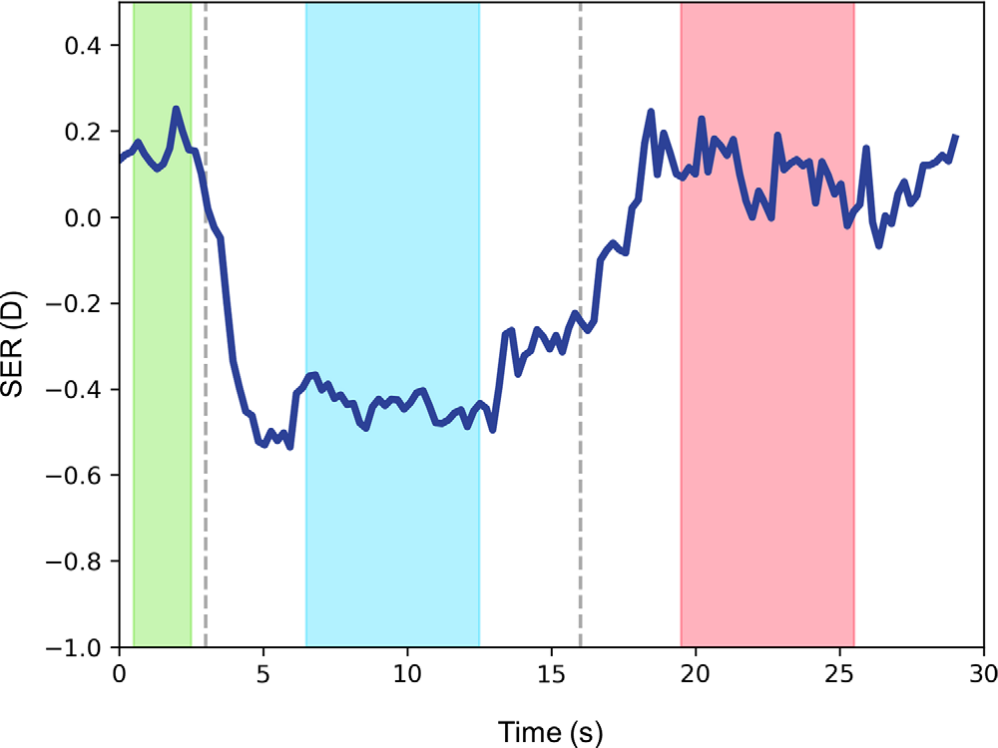
Analyses of SER and pupillary diameter. The *blue line* represents the raw data for the SER. Linear interpolation was applied to the missing ocular refraction and pupillary diameter values. The baseline values for the SER and pupillary diameter were calculated from the median values at 0.5 second and 2.5 seconds (*green bar*). The SER and pupillary diameter during fixation on the AR target were calculated from the median values at 6.5 seconds and 12.5 seconds (*blue bar*). The SER and pupillary diameter during fixation on the real target were calculated from the median values at 19.5 seconds and 25.5 seconds (*red bar*).

The baseline values for the SER and pupillary diameter were determined by the median values between 0.5 and 2.5 seconds (the measurement point was 10 points) after the start of measurement (Fig. 3). The SER and pupillary diameter during fixation on the AR target were determined by the median values between 6.5 and 12.5 seconds after the start of measurement. The SER and pupillary diameter during fixation on the real target were determined by the median values between 19.5 and 25.5 seconds after the start of measurement. Changes in the SER and pupillary diameter for the AR target were calculated as the difference in the values at baseline and at fixation on the AR target for each distance, whereas the changes in SER and pupillary diameter for the real target were calculated as the difference in the values at baseline and at fixation on the real target for each distance.

### Statistical Analysis

The differences in SER and pupillary diameter between the baseline values and those during fixation on the AR or real target for each distance were analyzed using the Wilcoxon signed-rank test with Bonferroni correction. Thus, the *P* values described hereafter were four times greater than the raw values. The changes in the SER and pupillary diameter during fixation on the AR and real target for each distance were analyzed using the Scheffé test. The relationships between the changes in the SER and pupillary diameter during fixation on the AR target were analyzed using a single linear regression analysis. The relationships between the baseline SER and pupillary diameter were also analyzed using single linear regression analysis, as were the relationships between the SER and UCVA in the Maxwellian display and LCD. All of the statistical analyses were performed using SPSS Statistics 26 (IBM, Chicago, IL, USA). *P* < 0.05 was considered statistically significant.

## Results

### Participant Characteristics

**Figure 4.**
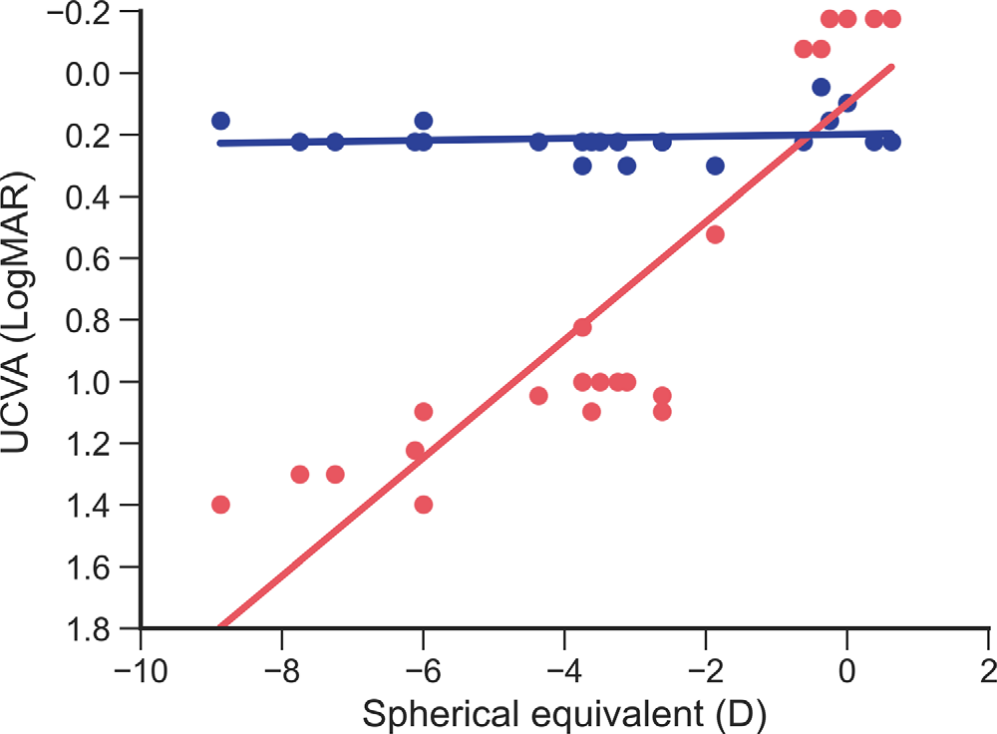
Comparison of UCVA between the Maxwellian display and trial frame in real space. The *blue* *points* and *red points* indicate the Maxwellian display and trial frame in real space, respectively. The *blue* *lines* and *red lines* indicate the regression lines. Maxwellian display: *R*^2^ = 0.032, *P* = 0.43; LCD: *R*^2^ = 0.640, *P* < 0.001.

The average visual acuity under soft contact lens correction was −0.138 ± 0.064 logMAR for the right eye and −0.147 ± 0.057 logMAR for the left eye. The average SER was −3.40 ± 2.68 D for the right eye and −3.59 ± 2.58 D for the left eye. The average angle of heterophoria was −1.9 ± 2.8 prism diopters (PDs) at near distance and −0.5 ± 1.4 PDs at far distance. All of the participants had a stereoacuity of 1.60 log arcsec (40 seconds equally). The participant characteristics are listed in Table 1. The UCVA using the Maxwellian display was not reduced by the participants’ SER (*R*^2^ = 0.032, *P* = 0.43). The UCVA using a trial frame in real space was reduced (*R*^2^ = 0.640, *P* < 0.001) (Fig. 4).

**Table 1.** Participant Characteristics

			BCVA (logMAR)		SER (D)		Ocular Deviation (PD)		
Participant Number	Age (y)	Sex	RE	LE	RE	LE	Near	Distant	Log Stereoacuity
1	21	F	−0.08	−0.18	−8.875	−9.75	−2	0	1.60
2	21	F	−0.18	−0.18	−6.00	−5.625	1	1	1.60
3	20	F	−0.08	0.00	−3.625	−3.75	−4	0	1.60
4	21	F	−0.08	−0.08	−7.75	−7.625	0	0	1.60
5	20	F	−0.08	−0.18	0.375	0.00	−2	0	1.60
6	21	F	−0.08	−0.18	−1.875	−1.5	2	1	1.60
7	20	F	0.00	0.00	0.625	0.625	−4	−2	1.60
8	21	F	−0.18	−0.08	−0.375	−1.00	−2	−1	1.60
9	20	F	−0.30	−0.18	0.00	−0.125	0	0	1.60
10	20	F	−0.18	−0.18	−6.00	−5.375	4	2	1.60
11	20	F	−0.08	−0.18	−7.25	−5.75	−6	−2	1.60
12	21	M	−0.18	−0.18	−3.75	−4.125	−2	−2	1.60
13	21	F	−0.18	−0.18	−0.625	−5.875	−8	−4	1.60
14	20	F	−0.08	−0.18	−3.75	−4.25	2	0	1.60
15	22	M	−0.08	−0.18	−6.125	−5.875	−4	−1	1.60
16	21	F	−0.18	−0.18	−3.25	−2.875	−4	0	1.60
17	21	F	−0.18	−0.18	−2.625	−2.625	−2	0	1.60
18	21	F	−0.18	−0.18	−2.625	−2.875	−2	0	1.60
19	21	F	−0.18	−0.18	−3.5	−2.625	−4	−2	1.60
20	21	F	−0.18	−0.18	−4.375	−4.625	−4	−2	1.60
21	21	F	−0.18	−0.18	−3.125	−3.125	2	2	1.60
22	20	F	−0.18	−0.08	−0.25	−0.25	−2	−2	1.60

The best-corrected visual acuity (BCVA) was measured with soft contact lenses in both eyes. Minus and plus signs in the ocular deviations indicate exodeviation and esodeviation of phoria, respectively.

LE, left eye; RE, right eye.

### Ocular Refraction

**Figure 5.**
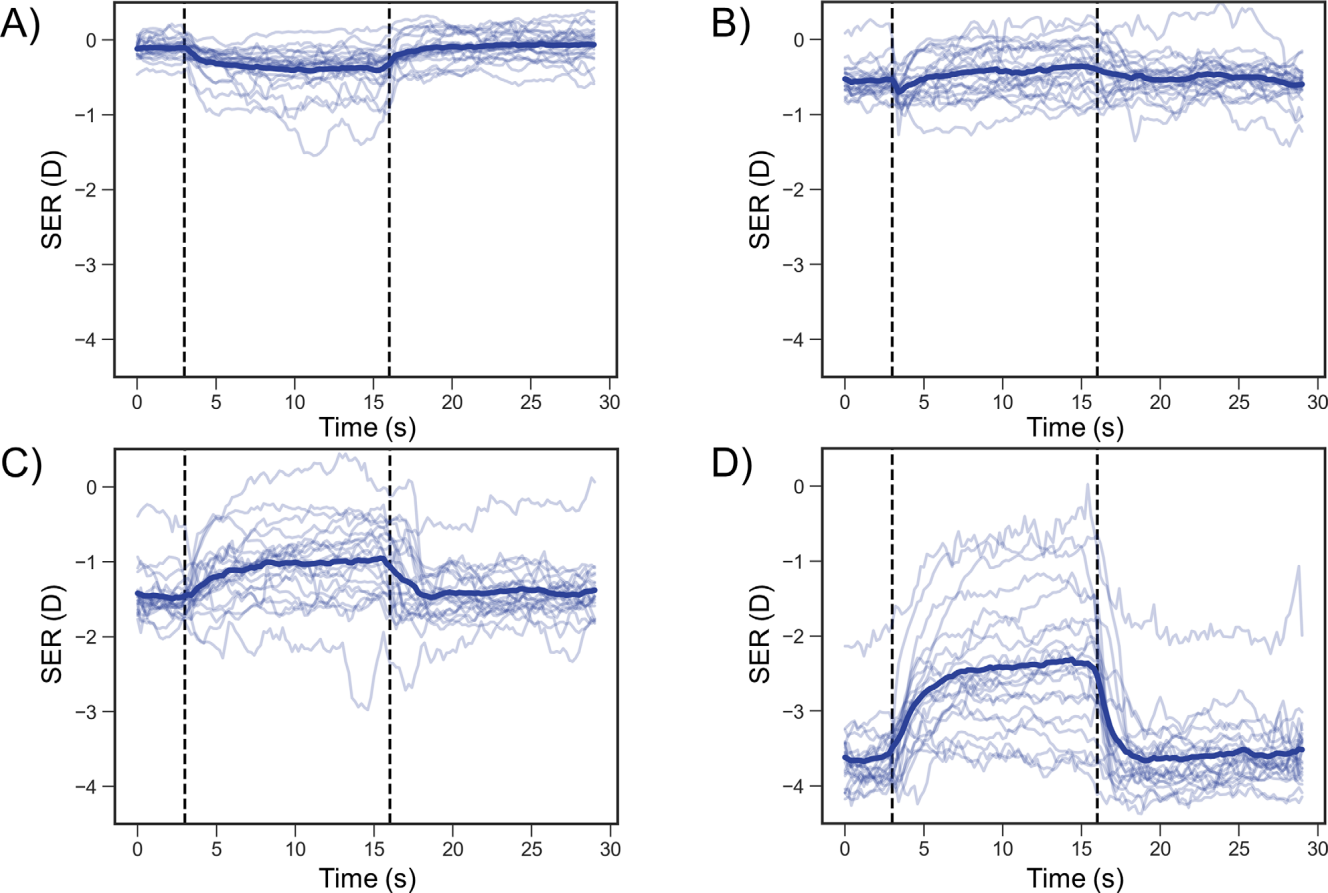
Individual and mean changes in SER. The *light* *blue lines* and *dark blue lines* indicate the individual and mean SER, respectively. The *y*-axis shows the SER, with larger negative values indicating greater myopia. The *vertical* *black interrupted lines* indicate when the target was switched from a real target to an AR target and back to the real target again. (**A**), (**B**), (**C**), and (**D**) indicate measurement distances of 5.0, 0.5, 0.33, and 0.2 meters, respectively.

Figure 5 shows individual and mean changes in the SER. The mean SER values are listed in Table 2. The SER was significantly more myopic during fixation on the AR target at 5.0 meters (−0.39 ± 0.27 D) compared to the baseline values (−0.11 ± 0.13 D; *P* < 0.001) (Fig. 5A). Moreover, the SER during fixation on the real target at 5.0 meters (−0.08 ± 0.18 D) was not significantly different from the baseline value (*P* = 0.85) (Fig. 5A). Additionally, the the SER did not significantly differ from the baseline values (−0.55 ± 0.22 D; AR vs. baseline, *P* = 0.68; real vs. baseline, *P* = 0.156) during fixation on the AR (−0.43 ± 0.31 D) and the real targets (−0.50 ± 0.25 D) at 5 meters (Fig. 5B).

**Table 2.** SER at Each Distance

				*P*	
Distance (m)	Baseline (D) Mean ± SD	AR Target (D) Mean ± SD	Real Target (D) Mean ± SD	AR vs. Baseline	Real vs. Baseline
5.0	−0.11 ± 0.13	−0.39 ± 0.27	−0.08 ± 0.18	<0.001	0.85
0.5	−0.55 ± 0.22	−0.43 ± 0.31	−0.50 ± 0.25	0.68	0.156
0.33	−1.47 ± 0.31	−1.04 ± 0.48	−1.40 ± 0.33	0.028	0.31
0.2	−3.64 ± 0.42	−2.44 ± 0.83	−3.60 ± 0.46	<0.001	>0.99

The same asterisk was used when measuring the values at baseline and the values during fixation on the real target. Statistical analysis was performed using the Wilcoxon signed-rank test with Bonferroni correction. The *P* values are four times greater than the raw values.

Moreover, the SER was significantly more hyperopic during fixation on the AR target at 0.33 meter (−1.04 ± 0.48 D) than at baseline (−1.47 ± 0.31 D; *P* = 0.028) (Fig. 5C). The SER during fixation on the real target at 0.33 meter (−1.40 ± 0.33 D) did not significantly differ from the baseline values (*P* = 0.31) (Fig. 5C). Moreover, the SER was significantly more hyperopic during fixation on the AR target at 0.2 meter (−2.44 ± 0.83 D) than at baseline (−3.64 ± 0.42 D; *P* < 0.001) (Fig. 5D). The SER during fixation on the real target at 0.2 meter (−3.60 ± 0.46 D) did not significantly differ from the baseline values (*P* > 0.99) (Fig. 5D).

**Figure 6.**
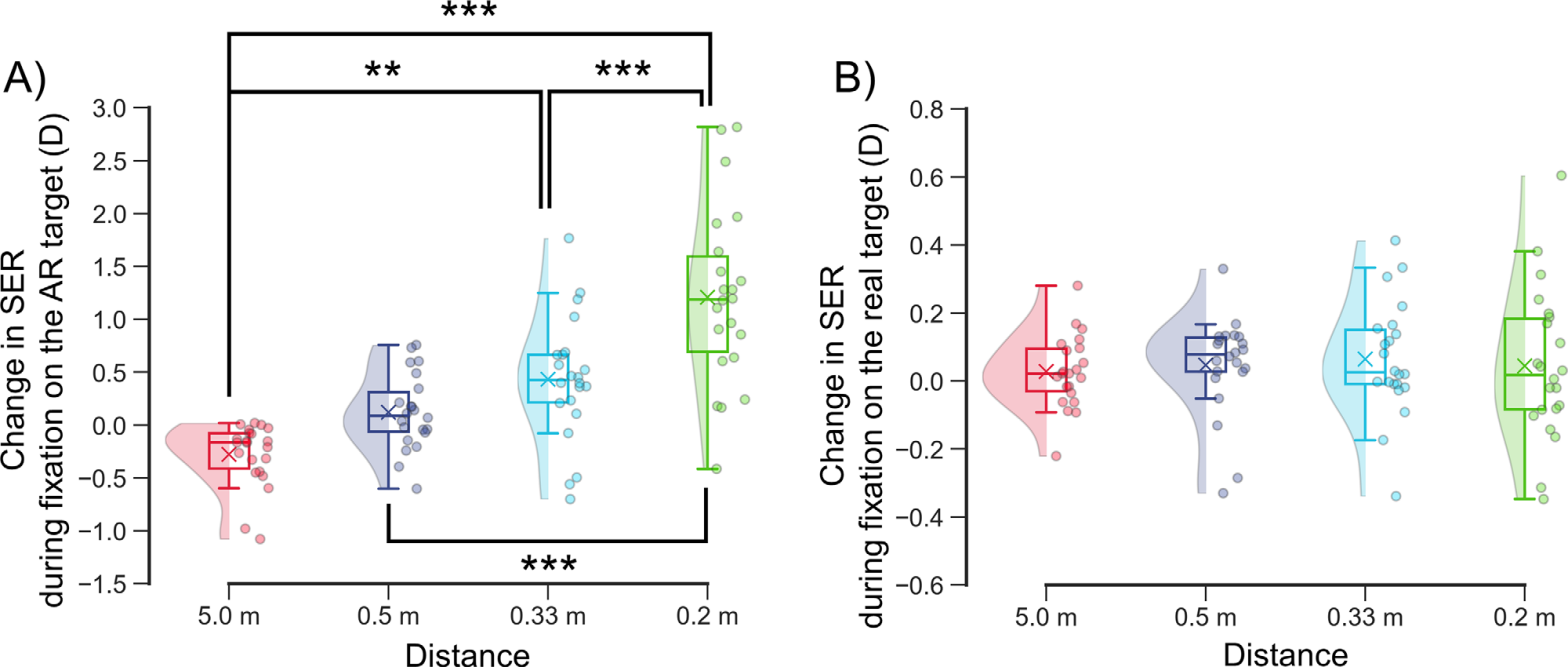
( **A** , **B**) Changes in SER during fixation on the AR target (**A**) and real target (**B**) at each distance. The raincloud plots show the raw data, data distributions, and key summary statistics, including medians, means (*crosses*), and interquartile ranges. Negative and positive values indicate hyperopic and myopic changes, respectively. (**A**) Changes in the SER during fixation on the AR target relative to the baseline SER at each distance. (**B**) Changes in the SER during fixation on the real target relative to the baseline SER at each distance. **P = 0.001, ***P < 0.001, Scheffé test.

During fixation on the AR target, the changes in the SER at 0.33 meter (0.43 ± 0.57 D) and 0.2 meter (1.20 ± 0.82 D) were significantly more positive than the changes at 5.0 meters (−0.28 ± 0.29 D; 0.33 vs. 5.0 meters, *P* = 0.001; 0.2 vs. 5.0 meters, *P* < 0.001) (Fig. 6A, Table 3). The change in SER at 0.2 meter was significantly more positive than the change at 0.5 meter (0.12 ± 0.35 D) and at 0.33 meter (*P* < 0.001) (Fig. 6A, Table 3). In contrast, during fixation on the real target, the change in SER did not significantly differ among distances (5.0 meters, 0.03 ± 0.10 D; 0.5 meter, 0.05 ± 0.14 D; 0.33 meter, 0.06 ± 0.16 D; 0.2 meter, 0.04 ± 0.22 D; *P* > 0.91) (Fig. 6B, Table 3).

**Table 3.** Changes in SER at Each Distance

Distance (m)	AR Target (D) Mean ± SD	Real Target (D) Mean ± SD
5.0	−0.28 ± 0.29	0.03 ± 0.10
0.5	0.12 ± 0.35	0.05 ± 0.14
0.33	0.43 ± 0.57	0.06 ± 0.16
0.2	1.20 ± 0.82	0.04 ± 0.22

The same asterisk was used when measuring the values at baseline and the values during fixation on the real target. The change in SER was calculated as the difference in the values at baseline and during fixation on the AR or the real targets for each distance.

### Pupillary Diameter

**Figure 7.**
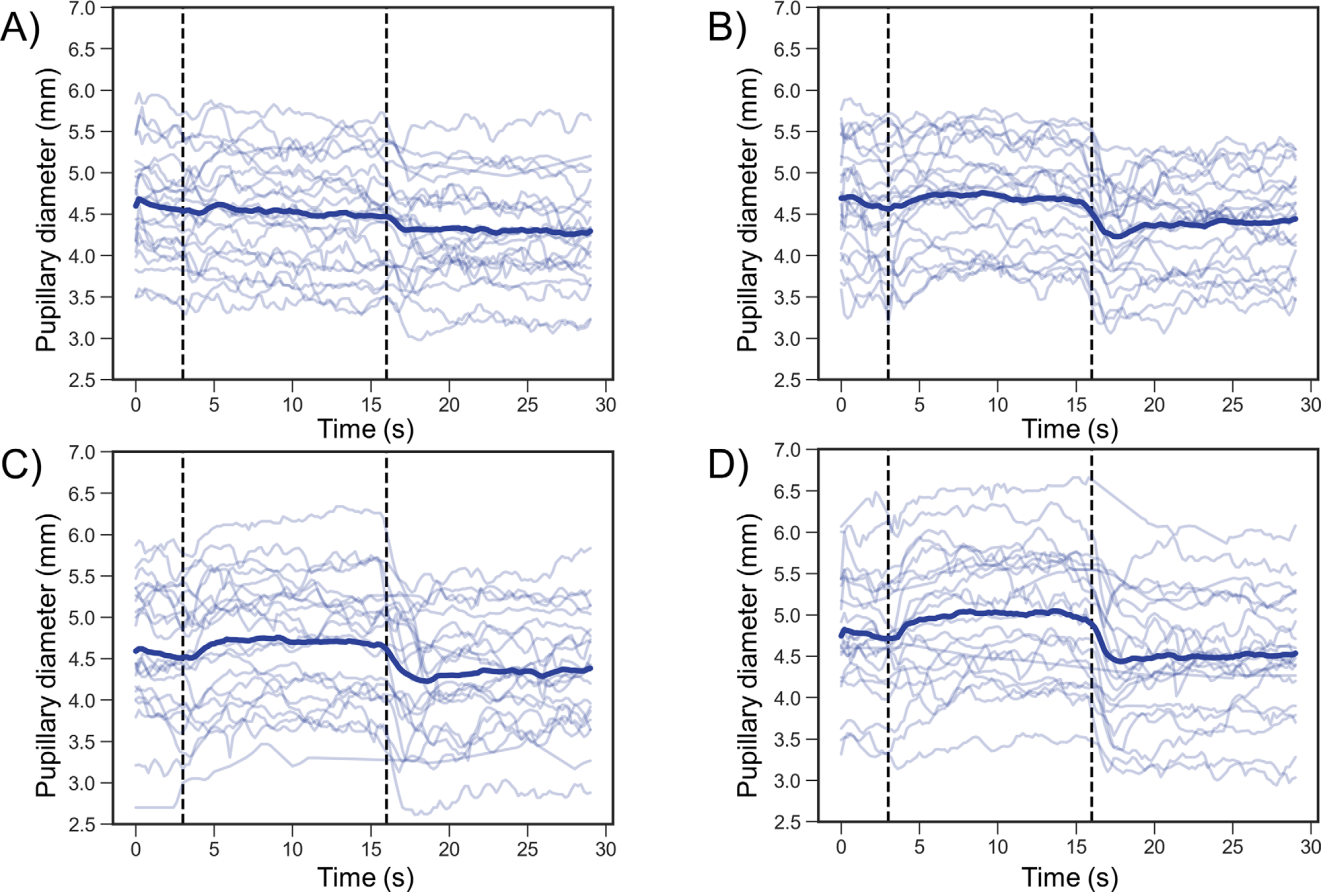
Individual and mean changes in pupillary diameter. The *light* *blue lines* and *dark blue lines* indicate the individual and mean pupillary diameters, respectively. The *y*-axis indicates the vertical pupillary diameter, and a smaller value indicates more miosis. The *vertical black interrupted lines* indicate when the target was switched from a real target to an AR target and back to the real target in that order. (**A**), (**B**), (**C**), and (**D**) indicate measurement distances of 5.0, 0.5, 0.33, and 0.2 meters, respectively.

Figure 7 shows the individual and mean changes in pupillary diameter. The mean pupillary diameters are summarized in Table 4. The pupillary diameter at a distance of 5.0 meters did not significantly differ during fixation on the AR (4.52 ± 0.64 mm) compared to the diameter at the baseline (4.59 ± 0.64 mm; *P* = 0.184) (Fig. 7A). During fixation on the real target at 5.0 meters (4.31 ± 0.62 mm), the pupillary diameter was significantly smaller than the diameter at baseline (*P* < 0.001) (Fig. 7A). Compared to its diameter at baseline (4.64 ± 0.67 mm; *P* = 0.132), the pupillary diameter did not significantly differ during fixation on the AR target at 0.5 meter (4.72 ± 0.62 mm) (Fig. 7B). The pupillary diameter was significantly smaller during fixation on the real target at 0.5 meter (4.38 ± 0.56 mm) compared to the diameter at baseline (*P* < 0.001) (Fig. 7B).

**Table 4.** Pupillary Diameter at Each Distance

				*P*	
Distance (m)	Baseline (mm) Mean ± SD	AR Target (mm) Mean ± SD	Real Target (mm) Mean ± SD	AR vs. Baseline	Real vs. Baseline
5.0	4.59 ± 0.64	4.52 ± 0.64	4.31 ± 0.62	0.184	<0.001
0.5	4.64 ± 0.67	4.72 ± 0.62	4.38 ± 0.56	0.132	<0.001
0.33	4.56 ± 0.81	4.72 ± 0.75	4.33 ± 0.69	0.012	0.004
0.2	4.77 ± 0.73	5.02 ± 0.78	4.49 ± 0.75	0.004	<0.001

The same asterisk was used when measuring the values at baseline and the values during fixation on the real target. Statistical analysis was performed using the Wilcoxon signed-rank test with Bonferroni correction. The *P* values are four times greater than the raw values.

Compared with the diameter at baseline (4.56 ± 0.81 mm), the pupillary diameter during fixation on the AR target at 0.33 meter was significantly greater (4.72 ± 0.75 mm; *P* = 0.012) (Fig. 7C). Compared with the diameter at baseline, the pupillary diameter during fixation on the real target at 0.33 meter was significantly smaller (4.33 ± 0.69 mm; *P* = 0.004) (Fig. 7C). The pupillary diameter was significantly greater during fixation on the AR target at 0.2 meter (5.02 ± 0.78 mm) than at baseline (4.77 ± 0.73 mm; *P* = 0.004) (Fig. 7D). Compared with the diameter at baseline, the pupillary diameter during fixation on the real target at 0.2 meter was significantly smaller (4.49 ± 0.75 mm; *P* < 0.001) (Fig. 7D).

**Figure 8.**
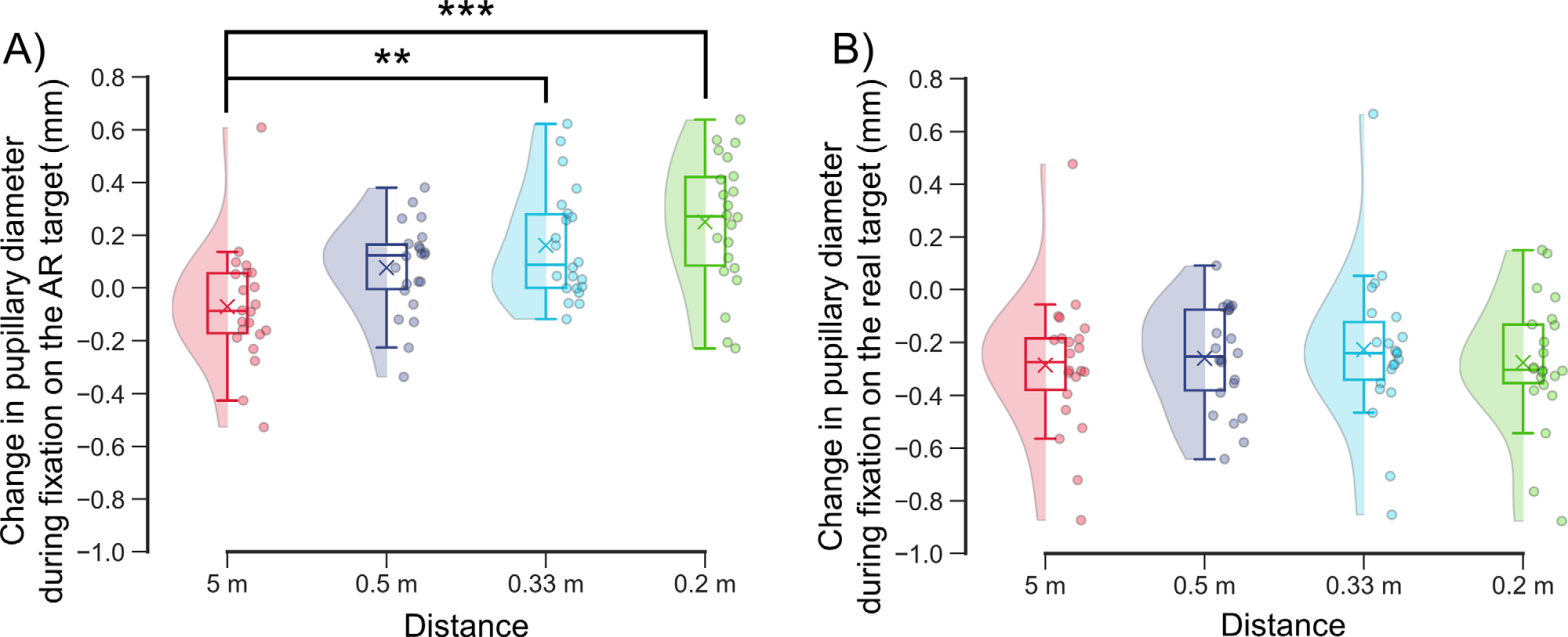
( **A** , **B**) Changes in pupillary diameter during fixation on the AR target (**A**) and real target (**B**) at each distance. The raincloud plots show the raw data, data distribution, and key summary statistics, including the medians, means (*crosses*), and interquartile ranges. The negative and positive values indicate pupillary constriction and dilation, respectively. (**A**) Changes in pupillary diameter during fixation on the AR target relative to the baseline pupillary diameter at each distance. (**B**) Changes in pupillary diameter during fixation on the real target relative to the baseline pupillary diameter at each distance. The baseline and real targets were the same. **P = 0.008, ***P < 0.001, Scheffé test.

During fixation on the AR target, the changes in pupillary diameter at 0.33 meter (0.16 ± 0.20 mm) and 0.2 meter (0.25 ± 0.24 mm) were significantly greater than the change at 5.0 meters (−0.07 ± 0.22 mm; 0.33 vs. 5.0 meters, *P* = 0.008; 0.2 vs. 5.0 meters, *P* < 0.001) (Fig. 8A, Table 5). The change in pupillary diameter at 0.5 meter (0.08 ± 0.17 mm) did not significantly differ from the constrictions at other distances (0.5 vs. 5.0 meters, *P* = 0.175; 0.5 vs. 0.33 meter, *P* = 0.64; 0.5 vs. 0.2 meter, *P* = 0.073) (Fig. 8A, Table 5). Additionally, the change in pupillary diameter did not significantly differ across all distances during fixation on the real target (5.0 meters, −0.28 ± 0.26 mm; 0.5 meter, −0.26 ± 0.19 mm; 0.3 meter, −0.23 ± 0.28 mm; 0.2 meter, −0.28 ± 0.24 mm; *P* > 0.91) (Fig. 8B, Table 5).

**Table 5.** Changes in Pupillary Diameter at Each Distance

Distance (m)	AR Target (D) Mean ± SD	Real Target (D) Mean ± SD
5.0	−0.07 ± 0.22	−0.28 ± 0.26
0.5	0.08 ± 0.17	−0.26 ± 0.19
0.33	0.16 ± 0.20	−0.23 ± 0.28
0.2	0.25 ± 0.24	−0.28 ± 0.24

The same asterisk was used when measuring the values at baseline and the values during fixation on the real target. The change in pupillary diameter was calculated as the difference in the values at baseline and at fixation on the AR or real targets for each distance.

**Figure 9.**
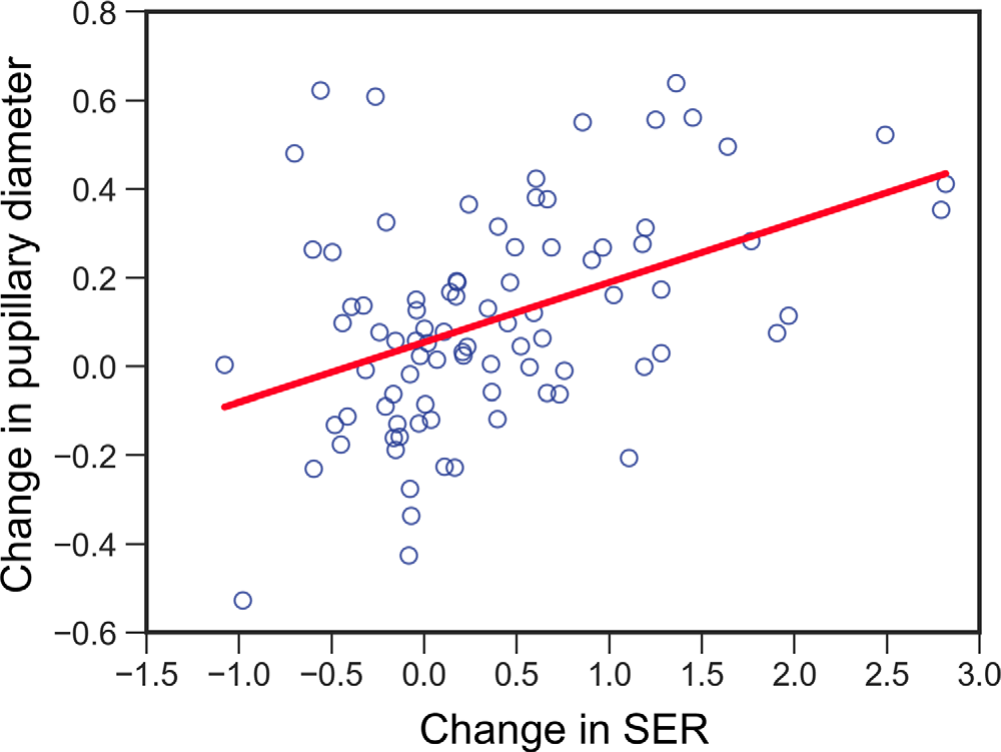
Relationship between changes in the SER and pupillary diameter during fixation on the AR target. The *red line* indicates the regression line. The *blue circles* indicate the changes in the SER and pupillary diameter at all distances. *R*^2^ = 0.187, *P* < 0.001.

In summary, the change in SER was significantly positively correlated with the change in pupillary diameter during fixation on the AR target (*R*^2^ = 0.187, *P* < 0.001) (Fig. 9). However, the change in SER did not correlate with the change in pupillary diameter during fixation on the real target (*R*^2^ = 0.010, *P* = 0.35). The baseline SER for each distance was not significantly correlated with the baseline pupillary diameter for each distance (*R*^2^ = 0.013, *P* = 0.30).

### Perceived Distance Between the AR and Real Targets

The number of participants who perceived the AR target to be in front of the real target at all distances was the highest (5.0 meters, 19 participants; 0.5 meter, 18 participants; 0.33 meter, 17 participants; 0.2 meter, 16 participants). This was followed by the number of participants who perceived the AR and real targets to be in the same position (5.0 meters, two participants; 0.5 meter, three participants; 0.33 meter, three participants; 0.2 meter, three participants). The number of participants who perceived the real target to be in front of the AR target was the smallest (5.0 meters, one participant; 0.5 meter, one participant; 0.33 meter, two participants; 0.2 meter, three participants).

## Discussion

This study evaluated the changes in ocular refraction of the SER and pupillary diameter during fixation on an AR target using a wearable Maxwellian display to prove that the Maxwellian display could not completely eliminate the accommodative response when used in real space. Overall, the SERs during fixation on the AR target increased in myopic power as the distance between the real target and the participant's eye decreased (Fig. 5, Table 2). SER was significantly more myopic during fixation on the AR target at 5.0 meter than was the baseline value during fixation on the real target (Figs. 5A, 6A; Tables 2, 3). The SER was significantly more hyperopic at 0.33 meter and 0.2 meter compared to the baseline value (Figs. 5C, 5D, 6A; Tables 2, 3), even though the AR target was perceived to be in front of the real target. Compared with the diameter at baseline, the pupillary diameter was significantly greater during fixation on the AR target at 0.33 meter and 0.2 meter (Figs. 7C, 7D, 8A; Tables 4, 5). Furthermore, the change in SER was significantly and positively correlated with the change in pupillary diameter during fixation on the AR target (Fig. 9). These findings demonstrate that accommodative and pupillary responses are induced during fixation on an AR image using a Maxwellian display in real space.

We measured the UCVA via a Maxwellian display and found that the UCVA was ≥0.3 logMAR (Fig. 4). Thus, we considered that the Maxwellian view was established in this experimental environment. The highest SER was −8.875 D (Table 1). The accommodative stimuli by the real target were within the depth of focus of the Maxwellian display at all examination distances. If the theory of Maxwellian view is correct, the SER during fixation on the AR target should return to baseline at a distance of 5.0 meter or should return to the SER during fixation on the AR target at a distance of 5.0 meter (Fig. 5, Table 2). However, our findings indicate that ocular refraction during fixation on the AR images, even if the Maxwellian view is achieved, still depends on the distance of the corresponding real-space object used as the reference.

The participants’ SERs were corrected with SCLs from −0.50 D to 0.00 D at 5.0 meters, although the lag of accommodation was greater at 0.33 meter and 0.2 meter than at 5.0 meters and 0.5 meter. These findings support the results of Labhishetty et al.,^30^ who simultaneously measured accommodation and visual acuity and reported that the lag of accommodation increased as accommodative stimuli increased in the autorefractometer. In the experiment of Labhishetty et al.,^30^ the WV-500 autorefractor (Grand Seiko, Tokyo, Japan), which is an older model of the WAM-5500 used in our study. The measurement principle of the WAM-5500 is the same as that of the WV-500. Therefore, we consider that the absolute values of the SER at 0.33 meter and 0.2 meter, which were measured in this study, are affected by the measurement bias of the autorefractometer. However, the SER during fixation on the AR target changes relative to the baseline and is not influenced by the measurement bias of the autorefractometer.

Compared to the pupillary diameter at baseline, the pupillary diameter at distances of 5.0 meters and 0.5 meter was not significantly different during fixation on the AR target (Figs. 7A, 7B, 8A; Table 4). The pupillary diameter during fixation on the AR target was significantly greater than the diameter at baseline at distances of 0.33 meter and 0.2 meter (Figs. 7C, 7D; Table 4). Furthermore, the change in pupillary diameter was significantly and positively correlated with the change in SER during fixation on the AR target (Fig. 9). These findings indicate that the pupillary diameter increased in parallel with the relative reduction in the accommodative response during fixation on the AR target.

Pupillary diameter is affected by light stimuli, as well as by accommodation.^31^^–^^34^ As mentioned before, the brightness of the Maxwellian display and LCD were adjusted to the same luminance of 180 cd/m^2^. Despite having the same luminance, the RETISSA, which focuses parallel light with narrow light fluxes on the pupil, is expected to have less attenuation of light intensity than the LCD. We hypothesized that the pupillary diameter during fixation on the AR target would be smaller than the diameter at baseline because the pupillary diameter is correlated with the light intensity at the retina.^31^^,^^33^^,^^35^ However, the results of our study did not substantiate our hypotheses. Our findings obtained using the RETISSA suggest that the pupillary diameter is more strongly affected by dilation due to a reduced accommodative response than by constriction due to light stimulation. The pupillary diameter is related to the visual field, and a decreased pupillary diameter causes narrowing of the visual field.^36^ We considered that the pupillary diameter would not be smaller during fixation on AR images with the RETISSA than during fixation on real targets; therefore, visual field constriction is less likely to occur.

In this study, the participants fixated on the asterisk displayed on the LCD both during the baseline measurement and during fixation on the real target. The stimulus amounts during the baseline measurement and during fixation on the real target were equal. However, compared with the diameter at baseline, the pupillary diameter was significantly smaller during fixation on the real target at all distances (Figs. 7, 8B; Tables 4, 5). The experiments were conducted at different distances (5.0, 0.5, 0.33, and 0.2 meters), sequentially. At the same distance, it took approximately 30 seconds to start the next measurement after the end of the previous measurement. After completing the measurement at one distance, it took approximately 1 minute to change the distance of the real target. The pupillary diameter was restored to the previous baseline value by the start of the next measurement. Thus, our findings suggest that transient pupillary constriction occurs when real objects are viewed after fixation on an AR image.

The transient pupillary constriction in this study was not related to the accommodative response because the change in pupillary diameter did not correlate with the change in the SER during fixation on the real target. Therefore, we believe that the transient pupillary constriction is due to light stimulation.^31^^,^^33^ As stated above, the light intensity at the retina is expected to be stronger for the RETISSA than for the LCD, even though the brightness measured by the luminometer is the same. Approximately 30 seconds are needed for pupillary constriction induced by light stimulation to recover after the light stimulus is turned off.^37^ Therefore, we speculated that the pupillary diameter was smaller than the baseline diameter when the participants were fixated on the real target because of the pupillary constriction caused by the light stimulus and recovery of accommodation after the switch from the AR to the real target. Moreover, we speculate that the transient pupillary constriction during fixation on the real target was removed by the time of the next measurement because the measurement time of the real target in this study was 13 seconds, and there was a 30-second interval before the next measurement. The transient pupillary constriction after fixation on AR images should be further studied by changing the measurement conditions due to safety concerns related to fixation on AR images in real space.

The baseline SER for each distance was not correlated with the baseline pupillary diameter for each distance. The baseline SER increased to myopic as the LCD position became closer, suggesting that an accommodative response was involved. The absence of pupillary constriction associated with the accommodative response in this study may be related to the age of the participants. Pupillary responses to accommodative stimuli are age dependent.^38^^,^^39^ Compared with middle-aged and older adults, younger individuals have less constricted pupils in response to accommodative stimuli, and their pupillary diameters barely change in response to accommodative stimuli of 3 to 5 D.

Most participants perceived the AR target to be closer than the real target. Earlier studies reported that the subjective distance perception for AR images was underestimated compared with that for real objects.^40^^,^^41^ Gagnon et al.^41^ reported that distance estimates increased with clutter and perspective cues. Our findings are in line with those reported by Gagnon et al., as our experiment was conducted with minimal distance cues and a dark background; however, the effect of luminance should also be considered. When presenting a virtual image in a real space, the luminance of the virtual image must be increased for the user to recognize the image. Increasing luminance improves the visibility of the AR image, which then covers the real object, and the user mistakenly perceives the virtual image as if it were in front of the real object.

An increase in luminance enhances accommodative and pupillary responses.^42^^,^^43^ Therefore, we speculate that the inconsistency between accommodation and subjective distance perception during fixation on the AR target is affected by the difference in luminance between the AR target and the real target. We did not evaluate the effect of the difference in luminance between the AR and the real targets in this study, as we were concerned that changing the adjustment position each time an AR image was viewed might cause visual fatigue.^14^ In the future, we plan to investigate the changes in accommodative and pupillary responses due to the difference in luminance between the AR and the real target.

The current study shows that the Maxwellian display does not ensure the complete absence of accommodation under real-world conditions. The accommodative response induced by the Maxwellian display depends on the distance of the real-world object used as the reference, which may lead to inconsistency between accommodation and subjective distance perception. Prior studies have evaluated subjective functions, including visual acuity, image recognition, and discomfort, under the assumption that the Maxwellian display is accommodation free.^21^^,^^22^^,^^44^ Therefore, we believe that it is necessary to reevaluate the effects of Maxwellian display use on human health under the assumption that an accommodative response is induced.

This study has several limitations. We were unable to identify a specific factor explaining the individual differences in the accommodative and pupillary responses during fixation on the AR target. We evaluated only accommodative and pupillary responses to stimulation of the central retina in this study. Stimulation of the para/perifoveal regions also induces accommodative and pupillary responses.^45^^–^^47^ We speculate that individual differences in the magnitude of response may depend on the extent and location of stimulation on the retina (i.e., the retinal shape). The RETISSA projects images directly onto the retina using a scanning laser; therefore, the amount of stimulation in the central fovea and para/perifoveal regions is almost the same. In future studies, we will investigate accommodative and pupillary responses when targets are presented to the fovea and para/perifoveal regions with a fixed stimulus magnification on the retina, considering the retinal shape of the participants.

## Conclusions

Fixating on AR images using a Maxwellian display induces accommodative and pupillary responses. The accommodative response depends on the distance of real objects. Overall, the Maxwellian display does not completely eliminate accommodation in real space.

## Supplementary Material

Supplement 1

Supplement 2
